# Novel *RAD50* variants lead to Nijmegen Breakage Syndrome–like disorder and unplanned recombinant human growth hormone treatment response

**DOI:** 10.3389/fendo.2026.1755251

**Published:** 2026-02-19

**Authors:** Yan Gong, MingYu Jiang, ShengNan Wu, Sheng Guo, YongFen Lyu

**Affiliations:** 1 Department of Endocrinology, Shanghai Children’s Hospital, School of Medicine, Shanghai Jiao Tong University, Shanghai, China; 2 Department of Molecular Laboratory, Shanghai Children’s Hospital, School of Medicine, Shanghai Jiao Tong University, Shanghai, China

**Keywords:** microcephaly, Nijmegen Breakage Syndrome-like disease, *RAD50* mutation, recombinant human growth hormone, short stature

## Abstract

**Background:**

Human *RAD50* gene mutations cause Nijmegen Breakage Syndrome-like disease, characterized by severe prenatal and postpartum growth retardation and microcephaly. It is very rare (less than 5 cases) with limited clinical data and treatment experience.

**Methods:**

Clinical information was collected on a boy with microcephaly and severe growth restriction, including birth history, clinical features, unplanned response to recombinant human growth hormone treatment, and five-year follow-up after growth hormone discontinuation. The child underwent trio-based whole-exome sequencing and Sanger sequencing to validate the mutation. Constructed variant plasmids were used for *in vitro* functional experiments and Western blots to evaluate the potential impact of the variants.

**Results:**

The boy was born at full term, with substantial growth retardation from infancy to early childhood. At the age of 4.5 years, the child with syndromic short stature was prescribed recombinant human growth hormone for height correction by junior resident physicians, with no genetic evaluation performed prior to treatment. After 5 years and 9 months of recombinant human growth hormone treatment, genetic analysis was done due to his evident microcephaly and distinctive facial features. Two novel variants (p.His1269Argfs2 and p.Ser844Asn) were identified in the *RAD50* gene. Western blotting revealed the presence of the Flag-tag and EGFP in RAD50-wt, but not in RAD50-mut (p.His1269Argfs2), indicating the frameshift mutation may markedly impair RAD50 protein expression or stability. Although recombinant human growth hormone significantly improved the patient’s growth rate (from -3.35 SD to -1.28 SD), these variants may serve as a potential molecular basis for Nijmegen Breakage Syndrome-like disease and could also increase the risk of tumor formation. Treatment with recombinant human growth hormone was discontinued when the patient was 10 years and 3 months old. A five-year follow-up showed no evidence of a tumor was observed; The 15-year-old patient's height ceased to increase and remained at 151.5 cm.

**Conclusion:**

In the current study, we identified and characterized a patient with two *RAD50* mutations. This report expands the clinical and genetic scope of *RAD50* mutations. For the first time, it describes the response to unplanned recombinant human growth hormone therapy and the risk of long-term tumors. This report is intended to raise clinical awareness of the risk-benefit balance of recombinant human growth hormone therapy for syndromic short stature.

## 1 Introduction

DNA damage/damage repair defect has been shown to significantly contribute to chromosomal instability, gene loss, and cancer development, resulting in severe health complications (1). Of the various forms of DNA damage, double-strand breaks (DSBs) are considered the most severe. The MRE11-RAD50-NBS (MRN) protein complex is recognized as the primary sensor that facilitates cellular responses to DNA damage, particularly DSBs (2). Mutations in all three components of the MRN complex have been reported to be associated with human autosomal recessive genetic diseases, including *MRE11* mutations causing ataxia-telangiectasia-like disorder (ATLD); *NBN* mutations causing Nijmegen Breakage Syndrome (NBS); and *RAD50* mutations causing Nijmegen Breakage Syndrome-like disorder (NBSLD) (3).

*RAD50* (OMIM: 604040) is a highly conserved DNA DSB repair gene located on chromosome 5q31, encoding a 153 kDa RAD50 protein (4). The RAD50 protein is a core component of the MRN complex and plays a crucial role in the process of DNA damage response. Human *RAD50* mutations lead to NBS-like disease, also called RAD50 deficiency, which is characterized by severe prenatal growth retardation and persistent postnatal growth restriction, congenital microcephaly, marginal to slight impaired intellectual development (5).

There are very few case reports (less than 5 cases) and limited clinical data and treatment experience for patients with NBSLD caused by *RAD50* mutations. As DNA damage/damage repair defects may be associated with cancer development (6), although growth restriction is an important clinical manifestation of NBSLD, there is currently no treatment data on recombinant human growth hormone(rhGH) therapy for growth retardation caused by *RAD50* mutations.

The present study delineates the characteristics of a child resulting from two novel *RAD50* gene mutations and the unplanned rhGH treatment response prior to a definitive diagnosis. Furthermore, the functional studies on the novel mutation were conducted.

## 2 Materials and methods

### 2.1 Clinical evaluation and genetic examination

Medical history and clinical manifestations were assessed in the patient, and physical examinations were performed. The genomic DNA of the proband family was extracted from the peripheral blood using the Qiagen blood extract kit, 51106. Trio-WES was performed for the family to search for phenotype-associated variants using the IDT xGen^®^ Exome Research Panel (IDT, USA), followed by sequencing on the HiseqX10 (Illumina, USA) platform, with an average depth of approximately >20X. Variants were classified for pathogenicity according to the ACMG (American College of Medical Genetics and Genomics) guideline (7).

### 2.2 Functional analysis - vector construction and transaction

The full-length cDNA fragment of human *RAD50*, including the Flag tag in the C-terminal region but excluding the stop codon, was subcloned into a pEGFP-N1 vector to create *RAD50*-wt. The c.3806-3807del mutation was constructed using a site-directed mutagenesis kit (C215-01, Vazyme) in the pEGFP-*RAD50* vector to create *RAD50*-mut. The two plasmids were then transfected into HEK-293T cells and harvested 72 hours later.

### 2.3 Functional analysis Western blot

The cells were lysed using RIPA buffer (P0013B, Beyotime) and the protein concentration was determined using a bicinchoninic acid assay (A55860, ThermoFisher). The sample was boiled for 10 minutes after the addition of 6× SDS loading buffer (P0015, Beyotime). The supernatant was separated using 10% SDS-PAGE with 20 µg of total protein per well, then transferred to a PVDF membrane (Bio-Rad, 1620174). The primary antibodies were incubated overnight at 4 °C and the secondary antibody for 1 hour at room temperature. The primary antibodies were Flag-tag (1:1000, 14793S, CST), EGFP (1:1000, 2956S, CST), and GADPH (1:1000, 5174S, ABclonal). HRP-linked goat anti-rabbit IgG (1:10000, 7074S, CST) was used as the secondary antibody.

### 2.4 Ethical approval

This study is a retrospective study. The research was performed in accordance with the principles of the Declaration of Helsinki. The study was approved by Shanghai Children’s Hospital Ethics Committee (2022R113-E01). Patients provided written informed consent to participate.

## 3 Results

### 3.1 Basic case presentation

The child was delivered at full term as the first child from non-consanguineous and healthy Chinese parents. The infant exhibited severe developmental delay during gestation, with a birth weight and height of 1.9 kg (−4.38 SD) and 46 cm (−3.24 SD), respectively. There was no record of any previous instances of asphyxia rescue. Furthermore, the record of the head circumference during the birth was not retrieved; however, microcephaly was noted at the time of birth. The height of his father and mother was recorded as 175 and 157 centimeters, respectively. Given that the height of his parents fell within the normal range, he had no familial history of growth retardation. Following a physical examination, it was determined that he exhibited a substantial growth retardation from infancy to early childhood. According to the parents’ reports, the child exhibited with mild learning difficulties. No history of recurrent infections was reported. He has a healthy younger sister, and no other family history was reported.

### 3.2 Height growth curve after rhGH treatment

**Figure 1:**
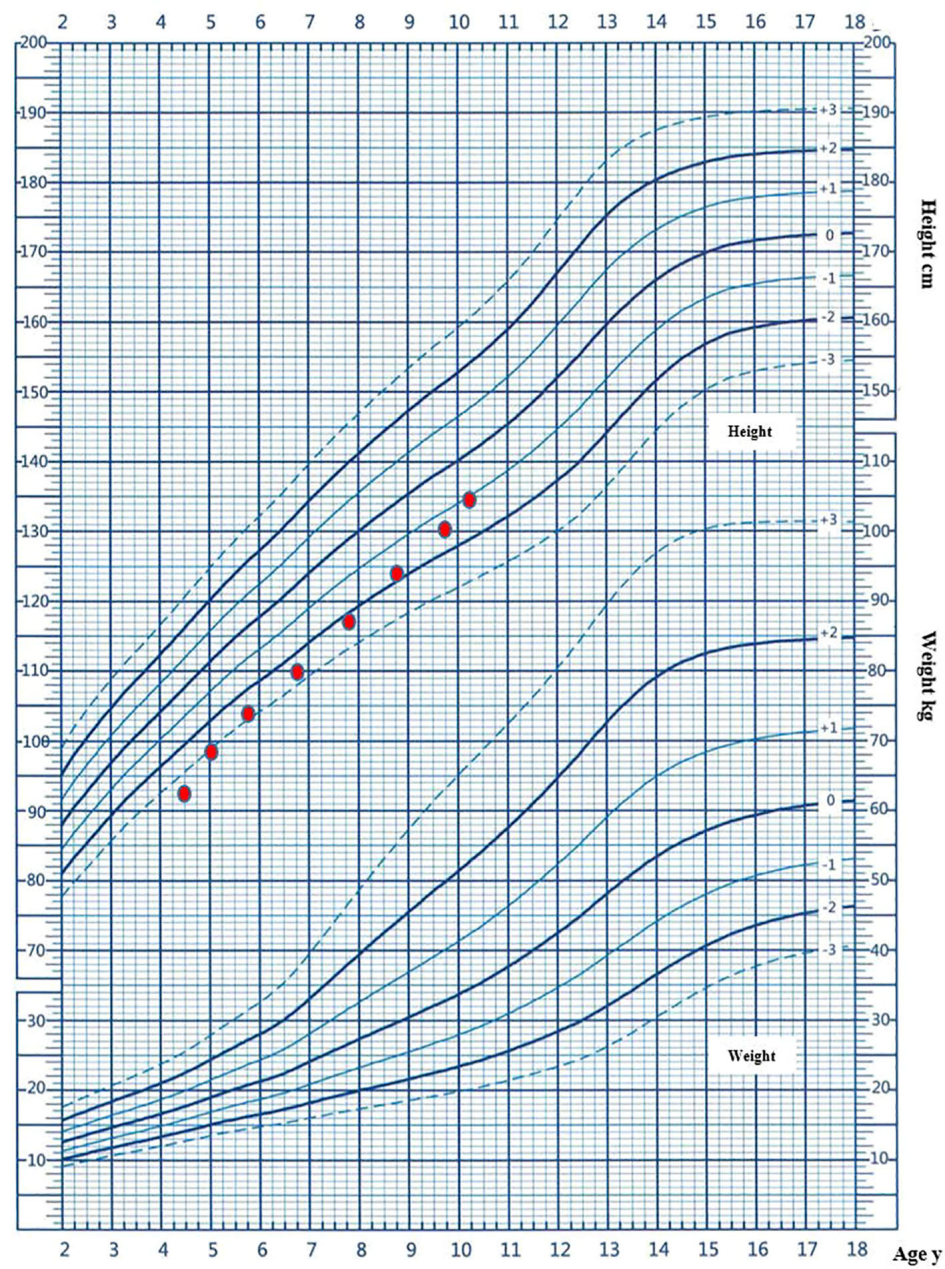
The patient’s growth curve following a period of five years and nine months of growth hormone treatment.

The child was admitted to the primary pediatric health department at the age of 4 years and 6 months, citing obvious growth disorders, characterized by a height of 93.9 cm (-3.35 SD) and a weight of 10.6 kg (-3.73 SD). He underwent a bone age examination, which showed 2 years and 6 months, significantly lagging behind in bone age, and a low level of the insulin-like growth factor 1 (IGF-1) level (80.07 ng/ml, -1.93 SD). Consequently, a growth hormone stimulation test was conducted, which revealed a peak growth hormone level of 10.60 ng/mL. No deficiency of growth hormone has been detected. Prior to genetic evaluation, rhGH treatment was initiated to address the patient’s short stature, since the diagnostic process for syndromic short stature had not yet been fully implemented in primary pediatric care. His initial growth hormone dose was 0.15 IU/kg/day, and his growth rate significantly increased after treatment. He was treated for a total of five years and nine months, achieving an average annual growth rate of 7.3 cm/year. During treatment, the dose fluctuated between 0.15 and 0.2 IU/kg/day according to the level of IGF-1. At the age of 10 years and 3 months, the child was 135 cm (-1.28 SD) tall, weighed 31.7 kg (-0.58 SD), and had a bone age of 11 years. The head circumference was 46.5 cm (-9.5 SD). The patient’s growth curve following a period of five years and nine months of growth hormone treatment is illustrated in **Figure 1**. During the 5-year and 9-month follow-up period, the child was regularly monitored for liver and kidney function, thyroid function, blood glucose, IGF1, and other abnormalities, and no adverse reactions were observed. Detailed information on *Follow-Up of the Child Before and After Treatment with Growth Hormone* is provided in **Supplementary Table 1**.

### 3.3 Clinical presentations and variants identified in the *RAD50* gene

Concurrently, the attending physician attained a more profound comprehension of the syndrome type of short stature and discerned that the child’s short stature, accompanied by distinctive facial characteristics and microcephaly, might be attributable to alternative genetic factors. In April 2020, the proband was referred to our center for the first time for genetic counseling.

**Figure 2:**
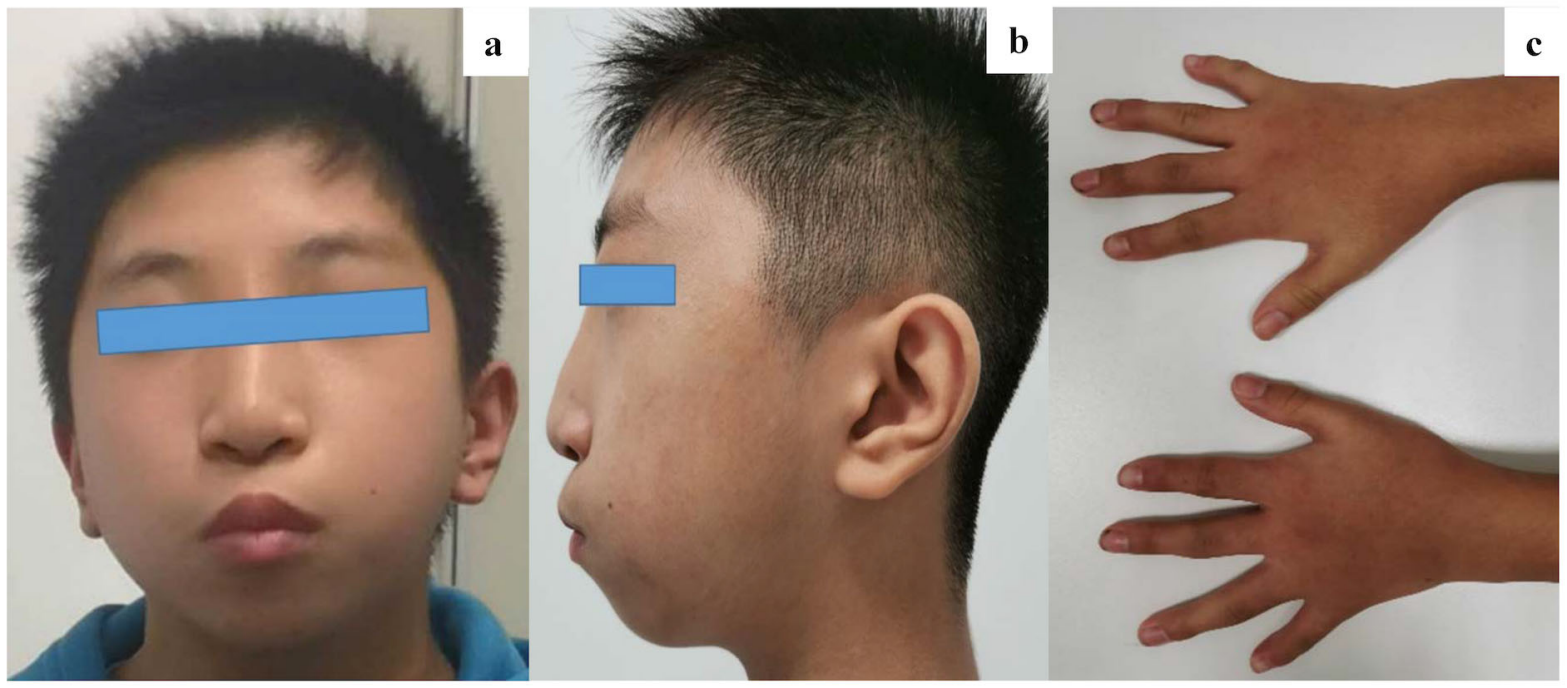
**(a, b)** Photographs of the facial features of the patient. Dysmorphic features include hypertelorism, receding forehead, long and narrow nasal bridge, small and prominent mouth, smooth and short philtrum, and micrognathia. **(c)** Both hands have the shortest middle phalanx in the little fingers.

When he arrived at our center, he had obvious facial deformities, including receding forehead, long and narrow nasal bridge, small and prominent mouth, smooth and short philtrum, and micrognathia (**Figures 2a, b**). Furthermore, the middle phalanx of the little finger of both hands was found to be short (**Figure 2c**).

**Figure 3:**
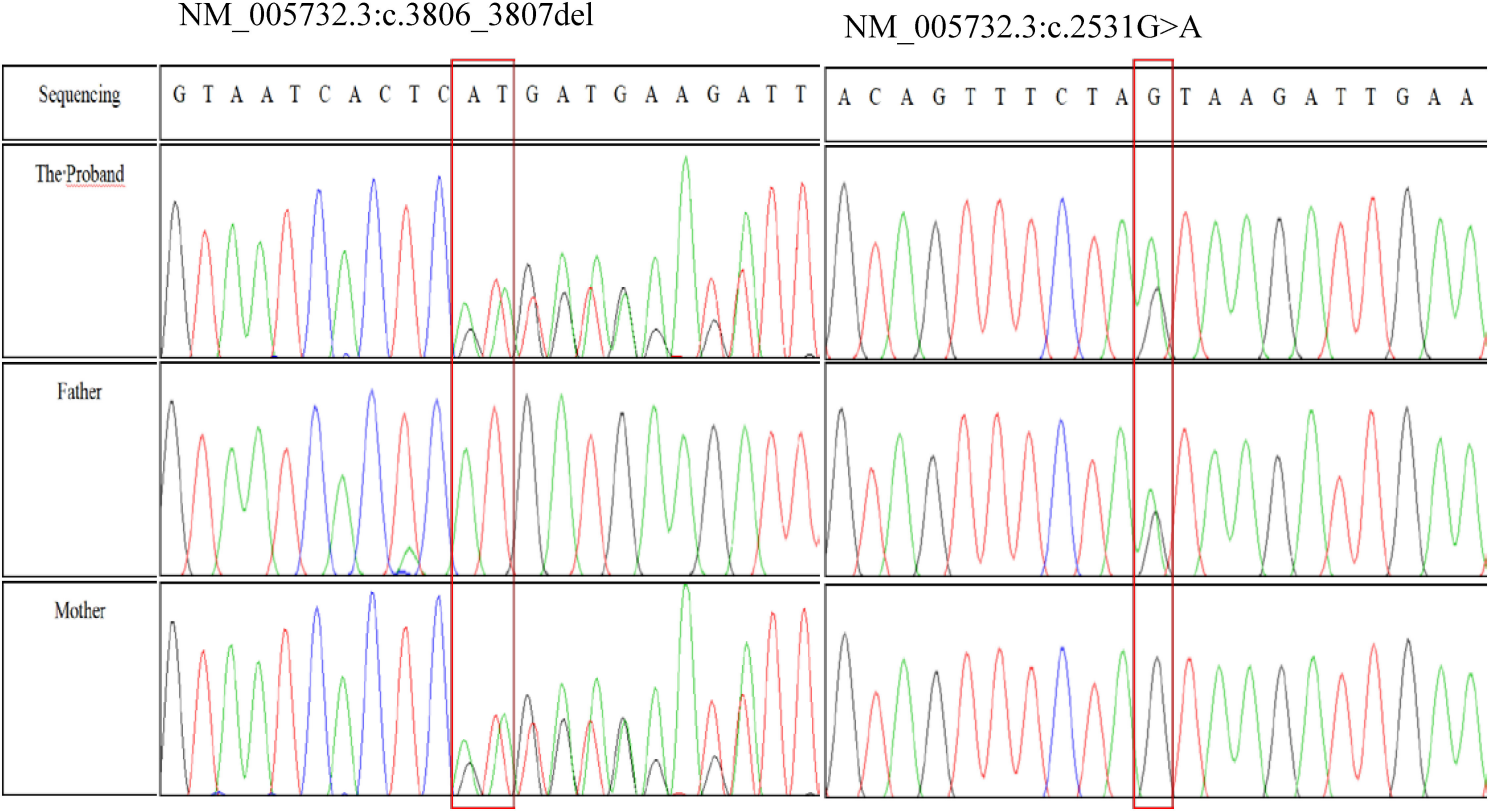
Sequencing of *RAD50* PCR products in the patient.

Trio-based whole-exome sequencing (trio-WES) was therefore performed, revealing two compound heterozygous variants in the *RAD50* gene: a frameshift variant, NM_005732.3:c.3806_3807del (p.His1269Argfs*2), inherited from the mother, and a missense variant, NM_005732.3:c.2531G>A (p.Ser844Asn), inherited from the father. Sanger sequencing confirmed the presence and parental origin of both variants (**Figure 3**).

### 3.4 Variants interpretation and functional analysis

The *RAD50* c.3806_3807del (p.His1269Argfs*2) variant was extremely rare in the gnomAD v4.1.0 database (allele frequency: 6.195 × 10^-7^) and has been submitted to ClinVar as a Variant of Uncertain Significance (VUS) in association with Nijmegen breakage syndrome–like disorder. This frameshift variant is located in the last exon of *RAD50* and is predicted to truncate the C-terminal region of the protein. However, PVS1 was not applied due to the limited evidence regarding the pathogenicity and disease mechanism of truncating *RAD50* variants in Nijmegen breakage syndrome–like disorder (e.g., insufficiently reported truncating cases and incomplete genotype–phenotype correlation). Overall, *RAD50* c.3806_3807del (p.His1269Argfs*2) was classified as a Variant of Uncertain Significance (VUS) under the ACMG/AMP framework.

The *RAD50* c.2531G>A (p.Ser844Asn) missense variant was also rare in gnomAD v4.1.0 (allele frequency: 1.244 × 10^-6^). In silico predictions supported a potential deleterious effect on protein function). The missense variant was inherited from the father and the frameshift variant from the mother, establishing the two variants *in trans*. Given that the frameshift variant on the other allele remains classified as VUS rather than pathogenic/likely pathogenic, the observation of in-trans configuration was considered supportive evidence for a recessive condition rather than definitive proof, and thus PM3_supporting was applied. Collectively, *RAD50* c.2531G>A (p.Ser844Asn) was also classified as a Variant of Uncertain Significance (VUS).

**Figure 4:**
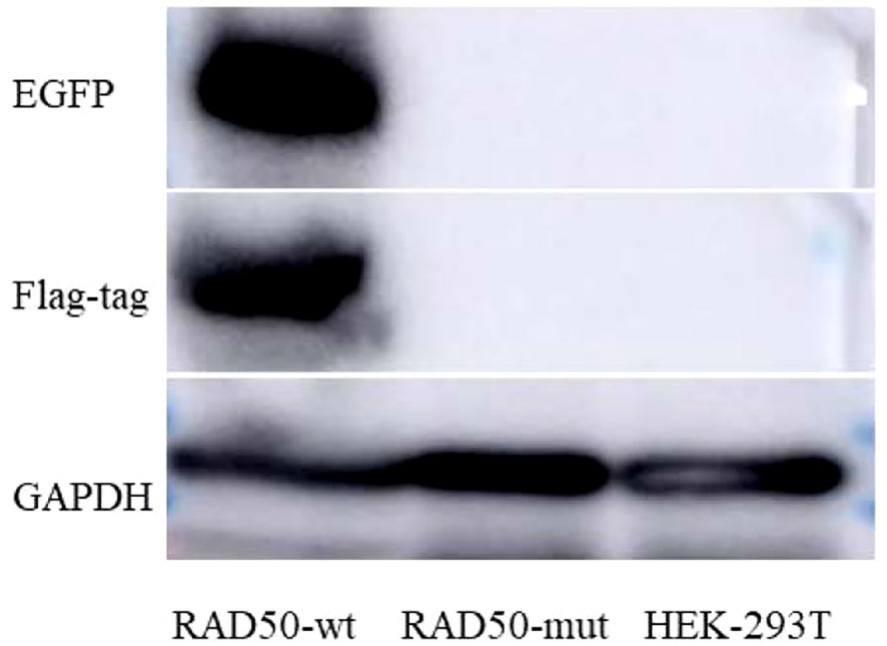
Western blotting analysis of wild-type and variant RAD50 expression. Western blotting revealed the presence of the Flag-tag and EGFP in RAD50-wt (wild type), but not in RAD50-mut(mutant RAD50 c.3806_3807del).

To investigate the potential impact of the *RAD50* c.3806_3807del variant on *RAD50* expression levels, western blotting revealed the presence of the Flag-tag and EGFP in *RAD50*-wt, but not in *RAD50*-mut (**Figure 4**). This finding indicates that the frameshift mutation c.3806-3807del (p.His1269Argfs * 2) may markedly impair RAD50 protein expression or stability. Taken together, the identification of two rare *RAD50* variants in trans, combined with the patient’s clinical features consistent with the Nijmegen breakage syndrome–like disorder spectrum and the observed reduction of RAD50 protein expression *in vitro*, suggests that these variants may represent a potential molecular basis for the disease. Additional functional studies and accumulation of further genetic evidence in independent patients will be required to clarify the pathogenic role of *RAD50* truncating variants in Nijmegen breakage syndrome–like disorder.

### 3.5 Follow-up after discontinuing rhGH therapy

As there is a possibility that DNA DSB repair defects caused by *RAD50* mutations may be related to cancer development, the use of rhGH was suspended immediately. He then received tumor markers, such as hCG, AFP, and CEA, all of which were within the normal range. An abdominal ultrasound was also performed to evaluate the morphology of various organs, and no malignant tumors were found. Subsequently, a period of follow-up spanning over five years was conducted, during which no evidence of tumor was observed in the child. His height has stopped growing, and he is currently 15 years old with a height of 151.5 cm. Due to concerns about radiation sensitivity, no examinations involving radiation (such as bone age or CT scans) were conducted.

## 4 Discussion

As previously mentioned, human RAD50 deficiency is categorized as an MRN-related disease. The MRN complex plays a vital role in DSB repair. We present the case of a Chinese boy who carries a compound heterozygous mutation in the *RAD50* gene and exhibits a distinctive phenotype. The identification of two rare *RAD50 v*ariants in trans, combined with the patient’s clinical features consistent with the Nijmegen breakage syndrome–like disorder spectrum and the observed reduction of *RAD50* protein expression *in vitro*, suggests that these variants may represent a potential molecular basis for the disease.

To date, only four individuals with NBSLD caused by *RAD50* mutations have been reported (5, 8–11). The comparisons of clinical and genetic characteristics of the patient from this study and the previous reports on *RAD50*-mutation patients are summarized in **Table 1**. Among patients with available data, the following characteristics were observed: intrauterine growth retardation; microcephaly at birth; short stature; progressive microcephaly; and specific facial features. They all exhibit normal sexual development and show no decline in nervous system; they only demonstrate mild learning disabilities. Some children exhibit symptoms of bone marrow failure and immune deficiency.

**Table 1:** Overview of clinical and genetic features of *RAD50* mutation.

Case characteristics	This report	Takagi et al., 2023 (11)	Ragamin etal., 2020 (10)	Chansel-Da Cruz et al., 2020 (5)	Barbi et al., 1991 (8); Waltes et al., 2009 (9)
Patient (n)	1	1	1	1	1
Sex	Male	Female	Female	Male	Female
Ethnicity	Chinese	–	Turkish	–	German
Mutation	c.3806_3807del c.2531G>A	c.248G > A c.1453G > T	c.2524G > A	c.2165dup c.3109_3111del	c.3277C>T c.3939A >T
Protein change and expression level	p.His1269Argfs*2 p.Ser844Asn	p.Arg83His p.Glu485X	p.Met800Phefs*7/low-level p.Val842Ile	p.Glu723 Glyfs*5 p.Glu1035del	p.Arg1093* p.X1313 TyrextX*66
Zygote state	Compound heterozygous	Compound heterozygous	Homozygous	Compound heterozygous	Compound heterozygous
Clinical manifestations at birth					
Birth weight (kg)	1.9(-4.38SD)	UN	1.52 (<−3.5 SD)	UN	1.83 (<−2.5 SD)
Birth head circumference (cm)	UN	UN	29.5 (<−3.5 SD)	UN	26.5 (<−6.5 SD)
Birth length (cm)	46(-3.24SD)	UN	36 (<−7.5 SD)	UN	41 (<−4 SD)
Microcephaly	+	+	+	+	+
Distinctive phenotype					
Growth retardation	+	+	+	+	+
Receding forehead	+	–	+	–	+
Long and narrow basal bridge	+	–	+	–	+
Small and prominent mouth	+	–	+	–	+
Prominent eyes	+	–	–	–	+
Smooth and short philtrum	+	–	+	–	+
Micrognathia	+	–	+	–	–
Short phalanges in both hands	+	–	+	–	+
Clinical manifestations at last examination					
Age at the time of last investigation	15 years	UN	15 years	7 years	23 years
Height (cm)	151.5 (<−3 SD)	UN	126.5 (<−5 SD)	108.5 (<3^rd^ percentile)	130 (<−4.5 SD)
Weight (kg)	34.5 (<−3 SD)	UN	25.3 (<−3.5 SD)	17.2 (<3^rd^ percentile)	29 (<−3 SD)
Head circumference (cm)	46.8 (−9.2SD)	UN	48.3 (<−5 SD)	48 (<3^rd^ percentile)	43 (−10 SD)
Sexual development	Normal	UN	Normal	UN	Normal
Neurological development	Normal	Normal	Normal	Normal	Normal
Intellectual disability	Mild	+	Borderline	Normal	Mild to moderate
Malignancies	–	–	–	–	–
Ataxia	–	–	–	–	Subtle unstable straight-line walk
Bone marrow failure	–	+	–	+	–

In MRN-related diseases, mutations in the MRE11 protein interfere with the apoptotic function of MRN complexes, leading to ATLD (12). The main characteristics of ATLD are progressive ataxia, neurodegenerative disorders and low susceptibility to cancer. The NBN mutation leads to Nijmegen breakage syndrome (NBS), which is typically characterized by microcephaly, growth retardation, immune deficiency and a high risk of tumors (13). Compared with individuals with ATLD or NBS, those with *RAD50* mutations do not exhibit significant neurological signs or symptoms, nor any signs of neurodegeneration. They only have mild learning disabilities, and it is unclear whether patients with NBSLD caused by *RAD50* mutations are at an increased risk of developing malignant tumors.

So far, no cases of malignant tumors caused by *RAD50* mutations in patients with NBSLD have been reported. However, it is worth mentioning that two cases of colon carcinoma and one case of lung carcinoma were found within the family of one patient with NBSLD caused by *RAD50* mutations (5). It has been reported that multiple different mutation sites of the *RAD50* gene have been detected in various cancers, including colorectal, lung, breast, and ovarian cancers, etc. (14–18). *RAD50* mutations lead to different manifestations (NBSLD or cancer), which may be related to the position of the mutation within the structure of the RAD50 protein. Different mutation positions can have different effects on the structure or function of the protein (3) (for example, C-terminal truncation is mainly manifested as NBSLD, whereas mutations affecting ATP hydrolysis are mainly manifested as cancer).

Our patient underwent unplanned growth hormone therapy without first undergoing genetic evaluation. Once the genetic diagnosis was underwent, the therapy was stopped immediately. He has been under observation for five years since discontinuation and there is currently no evidence of malignant tumors. However, due to the rarity of cases and the short follow-up period, we cannot rule out the possibility that patients with NBSLD caused by *RAD50* mutations are at an increased risk of malignancy. Patients with NBSLD caused by *RAD50* mutations should be regularly monitored for tumor occurrence. As molecular diagnostic approaches gain wider clinical acceptance, an expanding number of pediatric patients are anticipated to receive a diagnosis via whole exome sequencing in the coming years. Due to the DNA damage repair defect, repeated X-ray or CT scans should be avoided, and MRI or ultrasound scans should be used instead.

Against the backdrop of modern genomics, molecular diagnostic approaches enable the accurate identification of genetic etiologies underlying idiopathic short stature (ISS) in children. Although recombinant human growth hormone therapy has been approved for children with ISS in multiple countries (19). Clinical evidence has demonstrated that long-term growth hormone administration yields a moderate improvement in adult height among the majority of patients with ISS. Nevertheless, considerable uncertainties remain regarding the efficacy and safety of growth hormone therapy in a small subset of pediatric patients with rare syndromic short stature. Pediatric endocrinologists therefore require enhanced specialized training, redefine standardized diagnostic and therapeutic algorithms, deepen the understanding of the risk-benefit balance of growth hormone therapy for syndromic short stature, and establish a robust scientific rationale for the safety and efficacy of long-term growth hormone administration.

## 5 Conclusion

We present a patient with NBSLD caused by *RAD50* compound heterozygous mutations whose height increased following unplanned rhGH treatment. However, the *RAD50* mutation may increase the risk of tumor development, and further follow-up is needed to determine whether tumor formation occurs. Our report provides valuable data to help evaluate the unique clinical phenotype of NBSLD and improve pediatricians’ understanding of the risk-benefit balance of growth hormone in treating syndromic short stature.

## Data Availability

The original contributions presented in the study are included in the article/**Supplementary Material**. Further inquiries can be directed to the corresponding authors.
