# Supplementary material for: Novel RAD50 variants lead to Nijmegen Breakage Syndrome–like disorder and unplanned recombinant human growth hormone treatment response
Source: Front Endocrinol (Lausanne). 2026 Feb 19;17:1755251. doi: 10.3389/fendo.2026.1755251 (PMC12961580; doi:10.3389/fendo.2026.1755251)
Supplement: Supplementary file 1 [file DataSheet1.docx]

**Supplementary Material**

**Table 1 Follow-Up of the Child Before and After Treatment with Growth Hormone**

| **Age** | **Treatment Time** | **GH iu/kg/d** | **Height cm** | **Height SDS** | **Growth velocity cm/m** | **Weight kg** | **Bone age yr** | **IGF-1 ng/mL** |
| --- | --- | --- | --- | --- | --- | --- | --- | --- |
| 4y6m | Baseline | 0.150 | 93.0 | -3.50 | / | 10.6 | 2.5 | 80.07 (-1.93 SD) |
| 4y9m | / | 0.155 | 97.0 | -3.25 | 1.03 | 11.6 | / |  |
| 5y | 6m | 0.146 | 98.9 | -3.20 | 0.63 | 12.3 | / |  |
| 5y4m | / | 0.146 | 101.0 | -3.04 | 0.53 | 12.3 | / |  |
| 5y9m | 1y3m | 0.147 | 104.7 | -2.77 | 0.62 | 15.0 | / |  |
| 6y1m | / | 0.147 | 107.2 | -2.75 | 0.83 | 15.0 | / |  |
| 6y9m | 2y3m | 0.154 | 110.0 | -2.54 | 0.63 | 16.2 | 5.0 |  |
| 7y | / | 0.173 | 112.6 | -2.24 | 0.40 | 16.4 | / |  |
| 7y3m | 2y9m | 0.183 | 115.5 | -2.19 | 0.97 | 17.3 | / |  |
| 7y9m | / | 0.156 | 118.0 | -2.18 | 0.36 | 18.8 | / |  |
| 8y0m | 3y6m | 0.168 | 119.5 | -1.92 | 0.50 | 19.2 | / |  |
| 8y4m | / | 0.170 | 121.7 | -1.90 | 0.73 | 19.4 | / |  |
| 8y6m | 4y | 0.197 | 123.5 | -1.61 | 0.60 | 19.7 | / |  |
| 8y9m | / | 0.176 | 124.4 | -1.60 | 0.30 | 22.7 | / |  |
| 9y1m | 4y7m | 0.170 | 126.7 | -1.55 | 0.77 | 23.60 | / |  |
| 9y6m | / | 0.172 | 128.8 | -1.49 | 0.80 | 25.0 | / |  |
| 9y9m | 5y3m | 0.190 | 130.4 | -1.23 | 0.40 | 27.4 | / |  |
| 9y11m | / | 0.180 | 132.9 | -1.17 | 0.83 | 29.0 | / |  |
| 10y3m | stop | 0.160 | 135 | -1.16 | 0.53 | 31.7 | 11.0 |  |
